# Characterization of New Allergens from the Venom of the European Paper Wasp *Polistes dominula*

**DOI:** 10.3390/toxins13080559

**Published:** 2021-08-10

**Authors:** Johannes Grosch, Bernadette Eberlein, Sebastian Waldherr, Mariona Pascal, Clara San Bartolomé, Federico De La Roca Pinzón, Michael Dittmar, Christiane Hilger, Markus Ollert, Tilo Biedermann, Ulf Darsow, Maria Beatrice Bilò, Carsten B. Schmidt-Weber, Simon Blank

**Affiliations:** 1 Center of Allergy and Environment (ZAUM), Technical University of Munich, School of Medicine and Helmholtz Center Munich, German Research Center for Environmental Health, 80802 Munich, Germany; johannes.grosch@helmholtz-muenchen.de (J.G.); michael.dittmar@helmholtz-muenchen.de (M.D.); csweber@tum.de (C.B.S.-W.); 2 Department of Dermatology and Allergy Biederstein, Technical University of Munich, 80802 Munich, Germany; bernadette.eberlein@tum.de (B.E.); sebastian_waldherr@yahoo.de (S.W.); tilo.biedermann@tum.de (T.B.); ulf.darsow@tum.de (U.D.); 3 Immunology Department, CDB Hospital Clinic de Barcelona, Institut d’Investigacions Biomèdiques August Pi i Sunyer (IDIBAPS), Universitat de Barcelona, 08007 Barcelona, Spain; MPASCAL@clinic.cat; 4 Spanish Network for Allergy—RETIC de Asma, Reacciones Adversas y Alérgicas (ARADYAL), 28040 Madrid, Spain; 5 Immunology Department, CDB Hospital Clinic de Barcelona, Universitat de Barcelona, 08007 Barcelona, Spain; CSANBARTOLO@clinic.cat; 6 Allergy Section, Hospital Clinic de Barcelona, Clinical Medical Group, 08036 Barcelona, Spain; delaroca.federico@gmail.com; 7 Department of Infection and Immunity, Luxembourg Institute of Health (LIH), 4354 Esch-Sur-Alzette, Luxembourg; christiane.hilger@lih.lu (C.H.); markus.ollert@lih.lu (M.O.); 8 Department of Dermatology and Allergy Center, Odense Research Center for Anaphylaxis, University of Southern Denmark, 5000 Odense, Denmark; 9 Department of Clinical and Molecular Sciences, Polytechnic University of Marche, 60121 Ancona, Italy; m.b.bilo@staff.univpm.it; 10 Allergy Unit, Department of Internal Medicine, University Hospital of Ancona, 60126 Ancona, Italy

**Keywords:** allergen, Hymenoptera venom allergy, phospholipase A2, *Polistes dominula*, allergen cross-reactivity

## Abstract

Discriminating *Polistes dominula* and *Vespula* spp. venom allergy is of growing importance worldwide, as systemic reactions to either species’ sting can lead to severe outcomes. Administering the correct allergen-specific immunotherapy is therefore a prerequisite to ensure the safety and health of venom-allergic patients. Component-resolved diagnostics of Hymenoptera venom allergy might be improved by adding additional allergens to the diagnostic allergen panel. Therefore, three potential new allergens from *P. dominula* venom—immune responsive protein 30 (IRP30), vascular endothelial growth factor C (VEGF C) and phospholipase A2 (PLA2)—were cloned, recombinantly produced and biochemically characterized. Sera sIgE titers of Hymenoptera venom-allergic patients were measured in vitro to assess the allergenicity and potential cross-reactivity of the venom proteins. IRP30 and VEGF C were classified as minor allergens, as sensitization rates lay around 20–40%. About 50% of *P. dominula* venom-allergic patients had measurable sIgE titers directed against PLA2 from *P. dominula* venom. Interestingly, PLA2 was unable to activate basophils of allergic patients, questioning its role in the context of clinically relevant sensitization. Although the obtained results hint to a questionable benefit of the characterized *P. dominula* venom proteins for improved diagnosis of venom-allergic patients, they can contribute to a deeper understanding of the molecular mechanisms of Hymenoptera venoms and to the identification of factors that determine the allergenic potential of proteins.

## 1. Introduction

*Polistes dominula* (European paper wasp) populations are on the rise across Europe and the world. This highly invasive species, originating from the warmer regions of southern Europe and northern Africa, is one of the winners of climate change. For instance, sightings were not only reported in South Africa and the United States of America [1,2] but also in Germany, Poland and Ukraine [3,4,5]. Conquering new habitats almost always leads to contact with native species, in this case, humankind. An increase in sting incidents and, therefore, also in allergy to *P. dominula* venom (PDV) is to be expected. A similar phenomenon was observed for *Vespula vulgaris* (common yellow jacket), which is considered invasive in Alaska. Here, the new stinging insect led to an increase in encounters, including reports of fatal systemic reactions to the venom of *V. vulgaris* in the population [6,7].

Most territories are shared with species of the *Vespula* genus, such as *V. vulgaris* or *Vespula germanica* (German yellow jacket). Due to the similar morphology of some *Polistes* and *Vespula* species, convincingly identifying the culprit insect after a sting is hard for most patients and physicians [8,9]. In consequence, a thorough anamnesis and impartial diagnostic tools such as intradermal tests and component-resolved diagnostics (CRD) are of utmost importance to discriminate between allergies to different Hymenoptera species. Unfortunately, for some patients with sensitization to more than one venom, the current diagnostic approaches are not sufficient [10]. Available platforms allow the measurement of specific IgE (sIgE) to whole *P. dominula* and *V. vulgaris* venom (YJV) and to the single allergens Ves v 1 (Phospholipase A1B), Ves v 5 (Antigen 5) as well as Pol d 1 (Phospholipase A1) and Pol d 5 (Antigen 5), but there is no decisive marker allergen available [10] to safely distinguish these two venom allergies. To circumvent the problem, new and species-specific allergens might be added to the diagnostic panel. However, the number of potential allergens specific for PDV is decreasing [11]. The already known allergens from PDV are phospholipase A1 (Pol d 1), hyaluronidase (Pol d 2), dipeptidyl peptidase IV (Pol d 3), serine protease (Pol d 4) and antigen 5 (Pol d 5). Except for Pol d 4, all annotated PDV allergens have homologous proteins in other wasp venoms and are, therefore, (potentially) cross-reactive. Accordingly, an alternative approach to improve laboratory diagnostics is gaining in importance. By measuring the sIgE levels against a set of cross-reactive allergens, the most probable sensitizing insect can be detected in 69% of PDV/YJV-double-sensitized patients [12]. So far, only one set of cross-reactive proteins from PDV and YJV is part of daily diagnostic routine: Ves v 5 and Pol d 5, the venom antigen 5s [10]. Other proteins present in both venoms may be used to increase the diagnostic resolution. Potential sets of proteins are the well-established phospholipases A1 (Ves v 1 and Pol d 1) and the hyaluronidases (Ves v 2.0101, Ves v 2.0201 and Pol d 2), but also newly identified ones, such as dipeptidyl peptidases IV (DPPIV) or phospholipases A2 (PLA2) [11,13]. The set of Ves v 1 and Pol d 1 may especially be of great value for clinical practice, as 97–100% of Italian PDV allergic patients are sensitized to Pol d 1. The high prevalence of Pol d 1-sensitized patients makes it a stable and decisive marker for PDV allergy [14].

In addition, a second approach, CAP inhibition, is also indicated in patients with positive test results in skin tests and/or sIgE measurements for more than one Hymenoptera venom. Patient sera are preincubated with increasing doses of both venoms and homologous with heterologous inhibition are compared. Recent studies have shown the superiority of CAP inhibition over component-resolved diagnostics in determining the true sensitization in double-positive patients with *Polistes* spp. and/or *Vespula* spp. venom allergy [15,16]. The CAP inhibition benefits greatly from additional allergens that are available for clinical routine, since it is also based on the cross-reactivity of homologous allergens.

In a recent study, the venom proteomes of *P. dominula* and *V. vulgaris*/*germanica* were elucidated using mass-spectrometry [11]. The authors differentiate between two different groups of proteins in the venom: the ‘true venom components’ and the ‘venom trace molecules’ (household contaminants). In short, proteins of the ‘true venom component’ compartment either have signal sequences that cause the protein to be transported into the extracellular matrix, an established venom function, or homologous proteins that are known to cause allergies. On the other hand, ‘venom trace molecules’ have no signal sequence and are, therefore, constrained to their cell and tissue of origin. Out of the 100 proteins found in PDV, only 25 exhibit properties of a ‘true venom component’. Out of the 25 ‘true venom components’, 15 have not been part of any research regarding their allergenicity. Based on the then gathered data, in this study, three promising proteins—the immune responsive protein 30 (IRP30), vascular endothelial growth factor C (VEGF C) and phospholipase A2 (PLA2)—were chosen. As a secretory protein, IRP30 contributes to immunological responses in Hymenoptera and was further described in, e.g., *Apis mellifera*, *Bombus terrestris* and *Vespa mandarinia* [17,18,19]. In honeybees, IRP30 is expressed simultaneously with carboxylesterase in bacterial infection, but not during aseptic lesions [18].

The function of phospholipases in venoms is thoroughly described and well understood. Phospholipases belong to the enzyme class of hydrolases and as such cleave phosphoglycerides. Phospholipase A1 (PLA1) hydrolyzes the sn-1 position, yielding a free fatty acid and a phosphoglycerid, while PLA2 catalyzes the same reaction at the sn-2 position. Since phosphoglycerids are part of pro- and eukaryotic lipid bilayers—and therefore of cell membranes—venom phospholipases are known to disrupt cells.

One of the main mediators of Hymenoptera venom function is PLA1. This protein has not only been described as a major allergen, but also leads to, e.g., cell lysis, pore formation, platelet aggregation and hemolysis due to its enzymatic effects [20,21]. In addition to PLA1, PLA2 also plays a role in various venomous spiders, snakes and Hymenoptera (*A. mellifera, Apis cerana, Bombus terrestris*) [22,23,24]. PLA2 is a Ca^2+^-dependent, secreted phospholipase, which hydrolyzes membrane phospholipids of motor nerves and thereby induces cell lysis. Accordingly, they act as neuro- and myotoxins [23].

The purpose of VEGF C in venoms is not entirely clear. The PDGF family (PF00341) comprises several members with three different corresponding receptors—VEGFR-1, -2 and -3. Depending on the receptor binding, proteins of this family are either hemangiogenic, lymphangiogenic or both. VEGF Cs bind to VEGFR-2 and -3 and are, therefore, predominantly lymphangiogenic, but also play a role in the mitogenesis of blood epithelial cells [25,26,27]. This hemangiogenic function also induces an increase in the permeability of blood vessels and may contribute to the envenoming process [28]. VEGF Cs occur naturally as prepropeptides and, depending on the progression of cleavage, the binding affinity for VEGFRs is altered, i.e., increased [28].

The three proteins were recombinantly produced, biochemically characterized and tested for their respective allergenic potential using in vitro and ex vivo approaches.

## 2. Results

### 2.1. Selection and Characteristics of Putative PDV Allergens

Proteins included in this study were all identified by mass spectrometry in PDV [11]. Proteins were chosen based on the presence of a signal sequence for the extracellular matrix and/or if homologous proteins are described as Hymenoptera venom allergens.

The Toll-like receptor 8 (TLR8) identified in PDV (XP_015174910.1) does not exhibit a signal sequence and is therefore no ‘true venom component’. However, a soluble variant carrying a leading sequence, the so-called immune responsive protein 30 (IRP30) (AEN62318.1), is examined here, as it was described in other Hymenoptera venoms [17,18,19]. The role IRP30 takes on in PDV is not unambiguously resolved. As, in contrast to IRP30, TLR8 contains an extended N-terminus with an in silico-predicted transmembrane region, we conclude that IRP30 might be a soluble TLR8 variant. Soluble TLRs are already known for various TLR classes in mice and men, such as TLR2 and TLR4 [29,30,31]. IRP30 has a predicted molecular weight of about 30 kDa and no glycosylation sites.

Phospholipases are prominent venom proteins and are known to elicit allergic reactions in susceptible individuals. Beside the already annotated PLA1 from PDV (Pol d 1), two variants of a phospholipase A2 (PLA2) were found to be present in the venom. Some PLA2 proteins, such as honeybee venom (HBV) Api m 1, are known to be potent major allergens [32]. However, Api m 1 shows sequence identity of only 45% to the two PLA2 variants found in PDV. Proteomic data showed that there may be a PLA2 in *V. vulgaris* venom as well, but it has not yet been described as an allergen. Therefore, PDV PLA2 is not only a promising protein to be tested for allergenicity but might also be used to discriminate YJV and PDV allergy.

**Figure 1:**
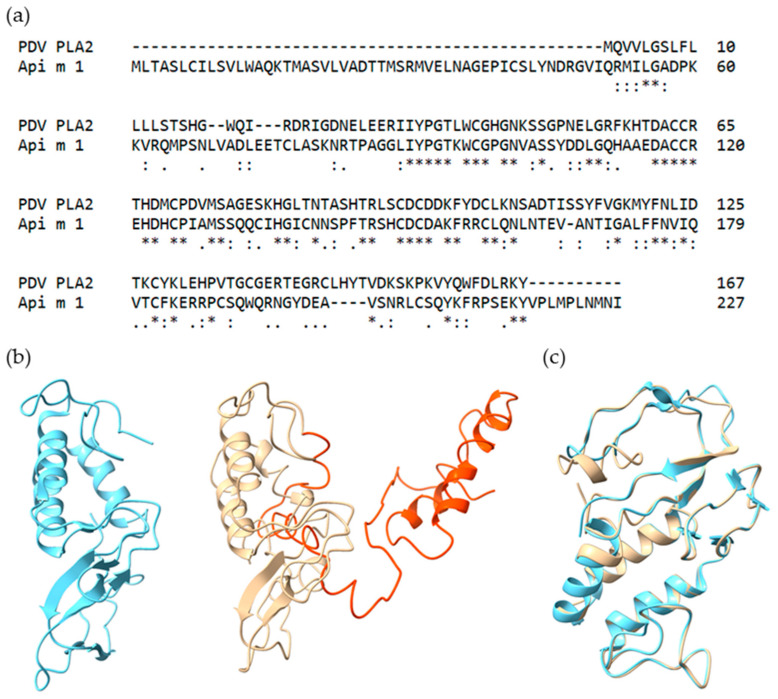
(**a**) Sequence alignment of *P. dominula* venom (PDV) phospholipase A2 (PLA2) and Api m 1. Sequence identity is at 44.6% (45/101) and sequence similarity at 62.4% (63/101). (**b**) Three-dimensional structure of Api m 1 (1POC) (blue) and PDV PLA2 (yellow) with its prolonged N-terminus (orange). (**c**) Superimposition of Api m 1 (blue) and PDV PLA2 (yellow) with truncated N-terminus.

The two variants of PDV PLA2 (227/229 residues identical, 2/229 missing) have two predicted glycosylation sites each (residues 84 and 142 and residues 82 and 140) and a theoretical molecular weight of 25 kDa. Figure 1a shows the sequence alignment of PDV PLA2 isoform X2 and Api m 1. The two sequences exhibit local similarity for 101 residues, of which 45 (44.6%) amino acids are identical, 63/101 (62.4%) similar and 1 missing (1%). Striking is the prolonged N-terminus of PDV PLA2 which, except for the signal sequence (residue 1–19), is of unknown function. No known protein domain, homologous superfamily or active site can be found within the residues 20–90.

Using the crystal structure of Api m 1 (1POC) as a template [33], the PDV PLA2 was homology modeled by Phyre^2^ Webserver. Figure 1b gives a structural overview of the two proteins. Again, the extended N-terminus (orange) is a remarkable feature of PDV PLA2, adding two α-helices linked by a flexible loop region to the basic structure shared by both proteins. Still, a high proportion of surface area is changed by the prolonged N-terminus compared to Api m 1. Superimposition of Api m 1 and PDV PLA2 yields a root mean square deviation of 0.461 Å between 105 pruned atom pairs. For the sake of clarity, the prolonged N-terminus of PDV PLA2 is not shown in Figure 1c. Despite a low sequence identity of 45% between Api m 1 and PDV PLA2, the tertiary structures of the two proteins show pronounced similarity.

Lastly, vascular endothelial growth factor C (VEGF C) was included in this study. VEGF C is part of the PDGF family (PF00341), which also comprises a candidate allergen from HBV, the PVF1. Russkamp et al. (2018) described PVF1 as an HBV component which is bound by sIgE from HBV-allergic patients (sensitization rate of about 40%) [34]. However, no basophil activation was observed using recombinant PVF1. The VEGF C examined here has a molecular weight of 36 kDa and shows two potential N-glycosylation sites (residues 119 and 261).

### 2.2. Recombinant Production and Biochemical Characterization of Putative PDV Allergens

Coding gene regions of all examined putative PDV allergens were amplified from PDV gland cDNA. To exclude reactivity of patients’ IgE to cross-reactive carbohydrate determinants (CCD) in immunoassays, the proteins were recombinantly produced using Sf9 insect cells. Sf9 cells provide proteins devoid of CCDs, while other N-glycosylations remain intact [35,36,37,38].

**Figure 2:**
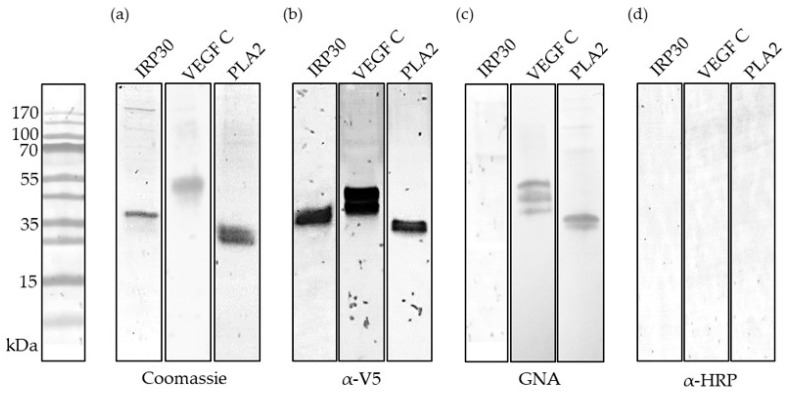
Analysis of recombinantly produced immune responsive protein 30 (IRP30), vascular endothelial growth factor C (VEGF C) and PLA2, stained by Coomassie Blue or detected via anti-V5 epitope antibody, *Galanthus nivalis* agglutinin (GNA) or anti-horseradish peroxidase antiserum (HRP). Displayed are strips of different SDS-PAGE gels and Western blots with a prestained protein ladder indicating the molecular weight in kDa.

After purification via Ni^2+^ affinity chromatography and molecular weight separation, the proteins were stained using Coomassie Blue or blotted onto NC membranes to detect fused V5-epitopes (Figure 2b). The predicted molecular weights for IRP30, PLA2 and VEGF C are 30, 25 and 36 kDa. Except for PLA2, which runs at approximately 25 kDa, the predicted molecular weights differ from those seen in gel electrophoresis. IRP30 and VEGF C show single bands at ~35 and ~45 kDa. Figure 2c,d further show all proteins and their respective interaction with *Galanthus nivalis* agglutinin (GNA) and anti-horseradish peroxidase antibodies (α-HRP). GNA binds to 1,2-, 1,3- and 1,6-mannose and thus can generally be used to detect N-glycosylation, while α-HRP is specific for α1,3-linked fucose, the CCD. As desired, none of the recombinantly produced proteins reacted with α-HRP, so that sIgE-reactivity due to CCDs can be excluded in further experiments. Detection with GNA leads to bands for VEGF C and PLA2 which confirm the presence of N-glycosylation on these proteins. Alone, IRP30 seems to be free of any N-glycosylation. These findings correspond to the in silico prediction of N-glycosylation sites.

### 2.3. sIgE Sensitization to Putative PDV Allergens

The presence of sIgE directed against recombinant IRP30, VEGF C and PLA2 in venom-allergic patients’ sera was assessed by ELISA. Sera of two different PDV-allergic patient populations, PDV-mono-sensitized (*n* = 16) and PDV/YJV-double-sensitized (*n* = 31), were included. Additionally, for PDV PLA2, as a potential marker allergen to discriminate PDV and YJV allergy, sera from YJV-allergic patients (*n* = 18) were added to the panel. Since homologous proteins to VEGF C and PLA2 are found in HBV, sIgE reactivity with these two proteins was further tested in an HBV-mono-sensitized population (*n* = 29) to assess potential cross-reactivity. Although IRP30 is known from several Apis species, it has not been described as part of their venom and was therefore not tested in HBV-allergic patients [39]. Neither IRP30 nor VEGF C are known venom components of *V. vulgaris* and accordingly have not been tested in YJV-allergic individuals [11].

**Figure 3:**
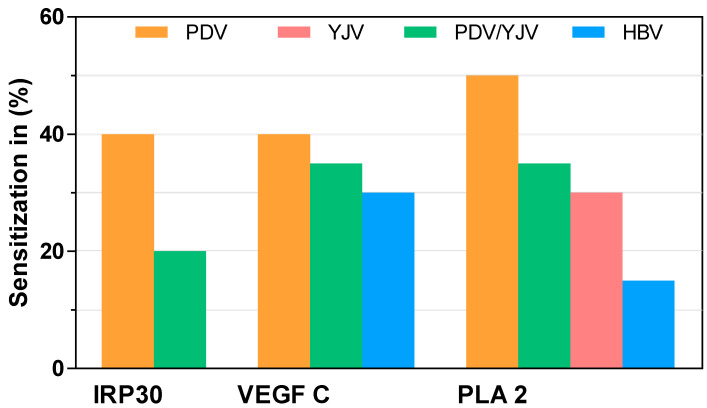
sIgE immunoreactivity of venom-sensitized patients to recombinant IRP30, VEGF C and PLA2 measured by ELISA in PDV mono- (*n* = 16), PDV/YJV (*n* = 31) double-, YJV (*n* = 18) mono- or HBV (*n* = 29) mono-sensitized patients. PDV, *P. dominula* venom mono-sensitized patients; PDV/YJV, *P. dominula/V. vulgaris* venom double-sensitized patients; YJV, *V. vulgaris* venom mono-sensitized patients; HBV, *A. mellifera* mono-sensitized patients.

Of the 16 sera from PDV-mono-sensitized patients tested, six demonstrated detectable levels of sIgE directed against IRP30, corresponding to 35–40% of patients. Double-sensitized PDV/YJV-allergic patients were less affected, with about 15–20% sensitization (5/31) (Figure 3 and Figure S1).

While 50% of PDV-mono-sensitized patients were sensitized to PLA2 (8/16), making it a potential major allergen, only 30–35% of patients sensitized to PDV and YJV tested positive for the presence of sIgE to PLA2 (10/31) (Figure 3 and Figure S1). Out of 18 mono-sensitized YJV-allergic patients, only five (25–30%) had detectable sIgE levels against PDV PLA2 (Figure 3 and Figure S1). In HBV-mono-sensitized patients, 4 out of 29 patients (10–15%) tested positive (Figure 3 and Figure S1). The four HBV-allergic patients above the cut-off were sensitized against the phospholipase A2 from HBV (Api m 1), as measured in Immuno-CAP (Table S1).

About 35–40% (4/16) of PDV-mono-sensitized patients, 30–35% (10/31) of PDV/YJV-double-sensitized patients (30–35%) and 25–30% (8/29) of HBV-mono-sensitized patients (25–30%) demonstrated sIgE reactivity to VEGF C (Figure 3 and Figure S1). 

### 2.4. Activation of Basophils from Allergic Patients by PDV PLA2 and HBV Api m 1

Since PDV PLA2 shows the characteristics of a major allergen and only exhibits minor reactivity in HBV- and/or YJV-allergic patient groups, basophil activation tests (BAT) were performed to verify the in vitro findings and to further examine its properties as a potential allergen. Patients were recruited from two different areas: 18 HBV- and/or YJV-allergic patients from Munich, Germany, (MUC) and 12 HBV-, YJV- and/or PDV-allergic patients from Barcelona, Spain (BCN) (Table S1). Sensitization against PDV cannot be completely excluded for the patients from Germany, since it is not part of clinical routine diagnosis. As a control, native Api m 1 (nApi m 1) was added to the BAT panel. The basophilic cells were stimulated using escalating concentrations of nApi m 1 or PDV PLA2 (1.6, 8, 40, 200 and 1000 ng/mL) and the increase in CD63 expression on the cell surface was measured.

**Figure 4:**
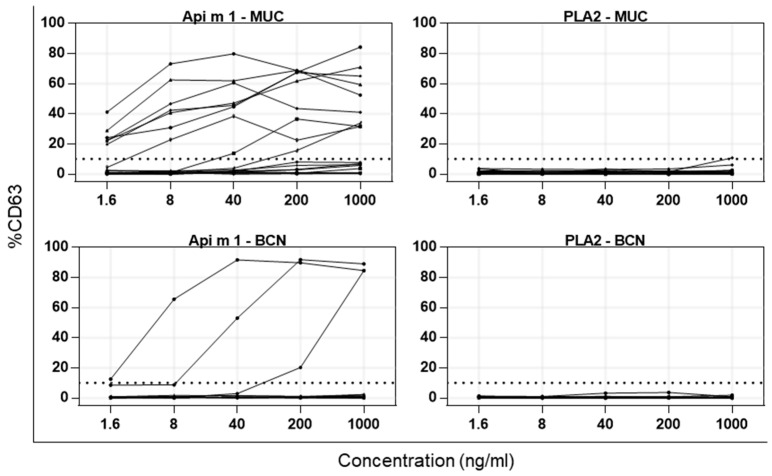
Increase in CD63 on basophilic cells after stimulation with Api m 1 or PDV PLA2. BAT for 18 patients from greater Munich (MUC) and 12 patients from greater Barcelona (BCN) area with diagnosed allergy or sensitization to HBV, YJV, HBV/YJV or PDV. Reactivity after stimulation with increasing doses of Api m 1 (left) and PDV PLA2 (right). Cut-off (dotted line) is at 10% of CD63 increase.

As was to be expected, reactivity to nApi m 1 can be observed in the patient groups from both regions (Table 1). In the MUC group, 9 out of all 18 patients tested show basophil activation upon stimulation, which corresponds to 50%. Of the BCN patients, 25% (3/12) are above the 10% cut-off (Table 1 and Figure 4). Patients without HBV sensitization and/or allergy were part of both groups. Excluding these patients leaves a total of 15 sera (13 positive for Api m 1 in Immuno-CAP (i208) (Table S1). Twelve show an increase in CD63 above 10% which translates to a sensitization rate of 87% to Api m 1 among HBV-sensitized and/or -allergic patients. Out of the 30 patients tested, only one patient’s basophilic cells were activated by stimulation with PDV PLA2 and only if administered at the highest concentration of 1000 ng/mL. As activation barely crosses the cut-off of 10% (10.75%), this was considered not relevant. Independent of the sensitization profile, the diagnosed Hymenoptera venom allergy and/or the area of recruitment, no basophil activation with PDV PLA2 was observed (Figure 4). The BAT results for individual patients are shown in Figure S2 (MUC) and Figure S3 (BCN).

## 3. Discussion

Discriminating PDV and YJV allergy may be achieved in the future by either adding new allergens to the diagnostic panel or measuring the sIgE titers against a set of cross-reactive allergens [12]. Based on the venom proteome of *P. dominula*, promising allergen candidates were chosen to be tested for their allergenicity. 

The secreted proteins IRP30, PLA2 and VEGF C from PDV were successfully cloned and recombinantly produced in Sf9 insect cells. Except for IRP30, all showed at least one N-glycosylation site in Western blot. The slight differences of about 10 kDa for IRP30 and VEGF C in the predicted and experimentally obtained molecular weight is likely due to the presence of a post-translational modification, such as N-glycosylations, or certain properties of the primary structure, such as a large proportion of acidic amino acids.

Sera of PDV-, PDV/YJV-, YJV- and HBV-allergic patients were used to determine sensitization rates in different Hymenoptera venom-allergic populations. IRP30 and VEGF C proved to be minor allergens, as sensitization in the PDV-allergic population was below 50% (25–40%). Of the same patients, 50% had sIgE directed against PLA2, rendering it a possible new major allergen. Since there are PLA2 proteins in HBV (Api m 1) and probably also in YJV, its cross-reactivity in the respective populations was assessed as well. Sensitization to PLA2 was 15% in HBV- and 30% in YJV-allergic patients.

However, while proteomic data show that there might be a PLA2 present in *V. vulgaris*/*V. germanica* venom, no genomic data has been published for either of the two species. A sequence comparison with PDV PLA2 to assess the sequence identities, including in silico predictions of, e.g., signal sequences or glycosylation status, is therefore challenging. A conclusive statement regarding whether the two PLA2s show significant similarities and, accordingly, cross-reactivity is not possible at the moment. 

In summary, the proteins IRP30 and VEGF C seem to be of minor relevance as allergens, although detectable levels of sIgE are present in essentially all patient populations included. The measured sensitization rates of 15 to 40% correspond to the classification of minor allergens which can only be used to a limited extent in CRD. PLA2 on the other hand shows characteristics of a major allergen (sensitization >50%) in its native patient population and limited cross-reactivity to its homologous proteins from HBV (10–15%) and YJV (25–30%).

To further analyze the allergenic potential of PDV PLA2, BATs were performed. BATs are considered a valuable tool for determining the clinical relevance of a sensitization, as it closely mimics the in vivo allergic reaction while avoiding the risk an allergen challenge imposes. It can not only be used in clinical routine for sophisticated diagnosis of allergies, but also in research to determine the allergenicity of candidate allergens in susceptible patients. Its main advantage over the classical approach of measuring sIgE against an allergen in experimental studies is the built-in downstream readout (the upregulation of CD63 on basophilic cells) that allows conclusions about an allergens’ ability to cross-link FcεRI on effector cells. BATs are frequently used in the context of venom allergies and have greatly improved diagnostics [40,41]. However, PLA2 did not lead to activation of basophilic cells in any of the 30 patients tested. False-negative results based on enzymatic activity of PDV PLA2 can be ruled out, as Api m 1 (HBV PLA2), which catalyzes the same reaction, leads to clear basophil activation in susceptible patients. This leads to the conclusion that PDV PLA2 does not seem to be cross-reactive to Api m 1 or its homolog from YJV. If PLA2 is a relevant PDV allergen is not entirely certain as several limitations have to be taken into account. The presence of sIgE in patients’ sera is not necessarily linked to the allergenic potential of a protein or the allergic status of the patient. For instance, sensitization against HBV or YJV is present in up to 23.1% and 31.7%, respectively, of the general population, while only 2.8% report symptoms more serious than local reactions [42]. Additionally, sIgE directed against C1q or PVF-1 from HBV was recently measured in vitro, but no basophil activation was demonstrated [34]. It was assumed that the downstream FcεR crosslinking on basophilic cells is somehow impaired and/or neutralizing antibodies of the IgG subclass were present. Taking the structural differences in PDV PLA2 and Api m 1 into account, it could be speculated that the prolonged N-terminus masks allergenic epitopes and/or sterically hinders the proper IgE-binding required for cross-linking. The relatively low sequence identity of 45% of Api m 1 to PDV PLA2 might influence the surface charge of PDV PLA2 and lead to impaired IgE binding. In addition, the patients diagnosed with PDV allergy who were included in BAT were not tested for their sIgE titers against PDV PLA2. Patients were recruited out of clinical routine; therefore, it cannot be ruled out that patients, by chance, were not sensitized to PDV PLA2. Even if well over 120 patients were recruited for the study, the decisive groups remain relatively small (~20 patients), which can have an impact on the calculated sensitization rates. Therefore, the data presented here are to be regarded as preliminary results that require further confirmation. In order to make a final statement as to whether PDV PLA2 is a major allergen, future research must assess the presence of neutralizing IgG antibodies in treated and untreated PDV-allergic patients and BATs must be carried out on a significant number of mono-sensitized PDV patients with sIgE to PDV PLA2 in order to confirm or refute its allergenic properties.

The proteomic data of the venom of PDV indicate that promising candidate allergens are scarce. After analyzing the sensitization against three true venom components (IRP30, PLA2 and VEGF C) and showing their minor relevance in terms of sIgE binding or ability to activate basophils, the chances have become even slimmer to find a reliable marker allergen to discriminate PDV and YJV allergies. Based on the here presented data and taking the current research into account [10], molecular diagnostics of Hymenoptera venom allergy will have to focus on known and characterized cross-reactive allergens such as the antigen 5 s, the phospholipase A1, the dipeptidyl peptidase IV and perhaps the hyaluronidases.

## 4. Materials and Methods

### 4.1. In silico Tools

ChimeraX v1.1 (RBVI, San Francisco, CA, USA, 2021) [43] was used to compare 3-dimensional protein structures. EMBOSS Matcher [44] allowed sequence comparison and analysis. GlycoEP web server [45] predicted the glycosylation status of the recombinantly produced proteins. For statistical analysis and graphs, GraphPad Prism 7.04 (GraphPad Software, San Diego, CA, USA, 2017) was used. InterProScan v5.49–83.0 [46] search yielded protein domains, homologous superfamilies and active sites. Phyre^2^ [47] gave a structural model of PDV PLA2 based on Api m 1 (1POC). ProtParam [48] was used to prognosticate physicochemical properties of the candidate allergens and SignalP4.0 [49] predicted the presence of leading sequences.

### 4.2. Cloning

Based on venom-gland-derived cDNA of *P. dominula*, protein-coding sequences were amplified by PCR with the following primer pairs. The reference sequences are given in square brackets: IRP30 [JN181874.1]/Toll-like receptor 8 (forward primer: GATCTCTAGAGAAGTTTGTAATGAGCGACAAGA; reverse primer: GATCGCGGCCGCTTATGAACGTTTTCTAATATTATTCACCT). VEGF C [XM_015329817.1] (forward primer: GATCTCTAGACAAAGTGATAATAGAAAGATCGATAAT; reverse primer: GATCGCGGCCGCTCATTTATATTCTGGATCATCCTTATG). PLA2 PA4-like isoform X2 [XM_015328329.1] (forward primer: GATCTCTAGAAGCGTGCTTGTTGCTGATACA; reverse primer: GATCGCGGCCGCTCATATATTCATATTCAATGGCATT).

IRP30 was cloned into the pFastBac Dual Expression Vector (Gibco, Carlsbad, CA, USA) using XbaI and NotI. The same restriction enzymes were used to clone PLA2 and VEGF C amplicons into the pFastBac1 vector (Gibco, Carlsbad, CA, USA). All constructs contain a 10-fold His-tag and a V5-epitope tag at the N-terminus. The generated recombinant donor plasmids were used for transformation of MAX Efficiency DH10Bac (Gibco, Carlsbad, CA, USA). Recombinant DH10Bac were cultured overnight at 37 °C in medium containing 50 µg/mL kanamycin, 10 µg/mL tetracycline and 7 µg/mL gentamycin sulphate solution in an incubator shaker. Cells were centrifugated and the pellet resuspended in 500 µL of resuspension buffer (25 mM Tris-HCL, pH = 7.9, 50 mM glucose, 10 mM EDTA, 100 µg/mL RNAse A). After adding 500 µL of lysis buffer (0.2 M NaOH, 1% (*w*/*v*) SDS), samples were incubated for 5 min at RT. A total of 500 µL of precipitation buffer (3 M potassium acetate (C_2_H_3_KO_2_), pH = 5.2) was added, and the samples inverted and put on ice for 10 min. Subsequently, samples were centrifugated for 10 min at 13,300 relative centrifugal force (rcf) at RT. A total of 800 µL of cold isopropanol (−20 °C) was added to 900 µL of the supernatant and incubated for 10 min on ice. After centrifugation at 13,300 rcf for 15 min at RT, the pellet was washed with 500 µL of 70% EtOH. The samples were centrifugated at 13,300 rcf for 5 min at RT, the supernatant discarded and samples dried for 5 min at RT. The yielded pellet was resuspended in 40 µL of ddH_2_O.

### 4.3. Generation of Recombinant Baculovirus and Recombinant Protein Production

As previously described [37,50,51], recombinant baculovirus was used to infect *Spodoptera frugiperda* insect cells (Sf9) (Thermo Fisher Scientific, Schwerte, Germany) to recombinantly produce secreted full-length proteins. In short, Sf9 cells were grown in Insect-XPRESS protein-free insect cell medium (Lonza, Basel, Switzerland) containing L-glutamine and 10 µg/mL gentamycin sulfate solution (Carl Roth, Karlsruhe, Germany) at 27 °C. Recombinant baculovirus was produced by transfection of Sf9 cells with 2 µg DH10Bac bacmid comprising the gene of interest. High virus titers were achieved by two rounds of virus amplifications. For protein expression, 400 mL of Sf9 cell suspension at a density of 1.5 × 10^6^ cells per ml was infected using 1 mL of the high titer virus stocks and incubated for 72 h at 27 °C and 110 rpm in a shaking incubator.

All proteins were purified using a nickel-chelating affinity matrix (HisTrap excel, GE Healthcare Life Sciences, Freiburg, Germany). PBS was used to wash the column and unspecifically bound proteins were pre-eluted with 45 mM imidazole in PBS. Proteins of interest were eluted by gradient elution (30–100%) with 300 mM imidazole in PBS. The samples were concentrated and diafiltrated (PBS, pH = 7.4).

### 4.4. SDS-PAGE and Western Blotting 

To confirm purity, recombinant proteins were separated using freshly casted 10% Tris-Tricine SDS-PAGE gels and stained with Coomassie Brilliant Blue G-250 (AppliChem, Darmstadt, Germany). For Western blots the proteins were blotted on nitrocellulose (NC) membranes and the unsaturated regions blocked with 4% of non-fat milk powder (AppliChem, Darmstadt, Germany) in PBS. Proteins were detected by mouse monoclonal anti-V5 antibody (0.2 µg/mL) (Thermo Fisher Scientific, Schwerte, Germany), biotinylated Galanthus nivalis agglutinin (10 µg/mL) (Vector Laboratories, Peterborough, United Kingdom) or rabbit polyclonal anti-HRP antiserum (1 µg/mL) (Thermo Scientific, Schwerte, Germany) and subsequently bound by anti-mouse IgG (0.4 µg/mL), ExtrAvidin (1:20,000) or anti-rabbit IgG (0.05 µg/mL) (Sigma, Taufkirchen, Germany) coupled to alkaline phosphatase. Using nitrotetrazolium blue chloride and 5-bromo-4-chloro-3-indolyl phosphate (AppliChem, Darmstadt, Germany) as substrates, the protein complexes were made visible. 

### 4.5. Patients

A total of 125 Hymenoptera venom-allergic patients were included in this study. For BAT, 30 patients were recruited, of which 18 came from greater Munich (Germany) and 12 from the greater Barcelona area (Spain). Additionally, 95 patients from Ancona (Italy) were included to determine the sIgE reactivity of patient sera. Criteria for participation was a diagnosed allergy to HBV, PDV, YJV or a combination of aforementioned venoms. Diagnosis was based on thorough anamnesis, clinical history of allergic sting reaction, positive intradermal skin test and/or sIgE levels as measured in the ImmunoCap system (Thermo Fisher Scientific, Uppsala, Sweden) to HBV (i1), PDV (i77), YJV (i3) and/or allergen components (i208, i209, i210, i211, i214, i215, i216, i217). The clinical data of all patients are given in Table S1.

All subjects gave their informed consent for inclusion before they participated in the study. The study was conducted in accordance with the Declaration of Helsinki, and the protocol was approved by the Ethics Committee of the Faculty of Medicine of the Technical University of Munich (5478/12), the ethical committee of the University Hospital of Ancona (N. 2016–0424) and the Comitè Ètic Investigació Clínica Hospital Clínic de Barcelona (HCB/2016/0361).

### 4.6. sIgE Reactivity of Patient Sera

To determine the sensitization rate in venom allergic patients, purified recombinant proteins were coated on 384-well microtiter plates (Nunc, Thermo Fisher Scientific, Ulm, Germany) at a concentration of 5–50 µg/mL in PBS and incubated at 4 °C overnight. The wells were saturated with 40 mg/mL fat-free milk powder (AppliChem, Darmstadt, Germany) in PBS at RT for 1 h and negative controls (i.e., no recombinant protein) with 40 mg/mL fat-free milk powder were added. After incubating with 20 µL of one part patient sera and two parts of PBS at 4 °C overnight, the wells were washed 4 times with PBS (pH = 7.4). Bound sIgE was detected using monoclonal alkaline phosphatase-conjugated anti-human IgE antibody (BD Pharmingen, Heidelberg, Germany) at a dilution of 1:1000. Wells were washed as described above, 50 µL of substrate solution comprising 100 mM Tris, 10 mM MgCl_2_*6H_2_O, 100 mM NaCl (pH 9.5) and 5 mg/mL 4-nitrophenylphosphate (AppliChem, Darmstadt, Germany) added and absorbance measured at a wavelength of 405 nm. To make a rough discrimination between positive (sensitized) and negative (non-sensitized) patients, an empirical but widely acknowledged cut-off calculation method was used. Three times the standard deviation of the mean absorbance of the negative controls was added to the mean and the resulting value increased by 10% to ensure that no systematic overestimation falsified the results [13,52,53]. The cut-off is shown in the graphs as a dotted line. Cut-offs were calculated per plate, protein and patient population. Instead of the exact calculated value, a range of 5 percentage points is given in the running text. Where possible, two independent experiments were combined into one data set and average values used for the calculation of sIgE reactivity.

### 4.7. Basophil Activation Test

Basophil activation tests (Flow CAST, Bühlmann Laboratories AG, Schönenbuch, Switzerland) were carried out as previously described [54]. In brief, venous blood was sampled and stored at 4 °C in EDTA tubes (1.6 mg EDTA/mL blood). Each recombinant protein to be tested, as well as native Api m 1 (Latoxan, Portes-lès-Valence, Frankreich), was solved in ddH2O and diluted in stimulation buffer to a final concentration of 1.6, 8, 40, 200 and 1000 ng/mL. As a positive control, a monoclonal anti-FcεRI antibody was added, while stimulation buffer alone served as a negative control. A total of 100 µL of the stimulation buffer comprising calcium, heparin and Il-3 (2 ng/mL) was mixed with 20 µL of staining reagent (anti-CD63-fluorescein isothiocyanate and anti-CCR3-phycoerythrin monoclonal antibodies) and 50 µL of venous blood. This mixture was subsequently added to the antigen dilution series and incubated for 25 min at 37 °C. Afterwards, to stop the stimulation, 2 mL of lysis buffer was added and incubated for 5 min at RT. Samples were centrifugated at 500 rcf for 5 min, the supernatant decanted and 300 µL of washing buffer added, before analyzing the cells by flow cytometry. Using anti-CCR3, basophilic cells were selected out of the total lymphocyte population. At least 300 (generally between 400 and 500) basophilic cells were analyzed and the upregulation of the activation marker CD63 on the cell surface calculated by the percentage of CD63 positive of total basophilic cells. As recommended by the manufacturer, the cut-off was set to 10% and added as a dotted line to the graphs.

## Figures and Tables

**Table 1:** Number of patients with basophil activation upon stimulation with either nApi m 1 or PDV PLA2. In total, 30 patients were included for basophil activation tests (BAT), 18 from greater Munich (MUC) and 12 from greater Barcelona (BCN) area.

	nApi m 1	PDV PLA2
**All patients (30)**	12	1
**MUC patients (18)**	9	1
**BCN patients (12)**	3	-
**HBV sensitized (15)**	12	1
**PDV sensitized (7)**	1	-
**YJV sensitized (24)**	7	1

## Data Availability

All data generated or analysed during this study are included in this article and its Supplementary Material files.

## Supplementary Materials

The following are available online at https://www.mdpi.com/article/10.3390/toxins13080559/s1, Table S1: Clinical data of patients, Figure S1: In vitro sIgE reactivity, Figure S2: BAT results for patients from Munich, Figure S3: BAT results for patients from Barcelona.
