# Supplementary material for: Characterization of New Allergens from the Venom of the European Paper Wasp Polistes dominula
Source: Toxins (Basel). 2021 Aug 10;13(8):559. doi: 10.3390/toxins13080559 (PMC8402607; doi:10.3390/toxins13080559)
Supplement: Supplementary file 1 [file toxins-13-00559-s001.zip › supplement.pdf]

Supplementary Materials: Characterization of New Allergens from the Venom of the European Paper Wasp *Polistes dominula*

Johannes Grosch, Bernadette Eberlein, Sebastian Waldherr, Mariona Pascal, Clara San Bartolomé, Federico De La Roca Pinzón, Michael Dittmar, Christiane Hilger, Markus Ollert, Tilo Biedermann, Ulf Darsow, Maria Beatrice Bilò, Carsten B. Schmidt-Weber and Simon Blank

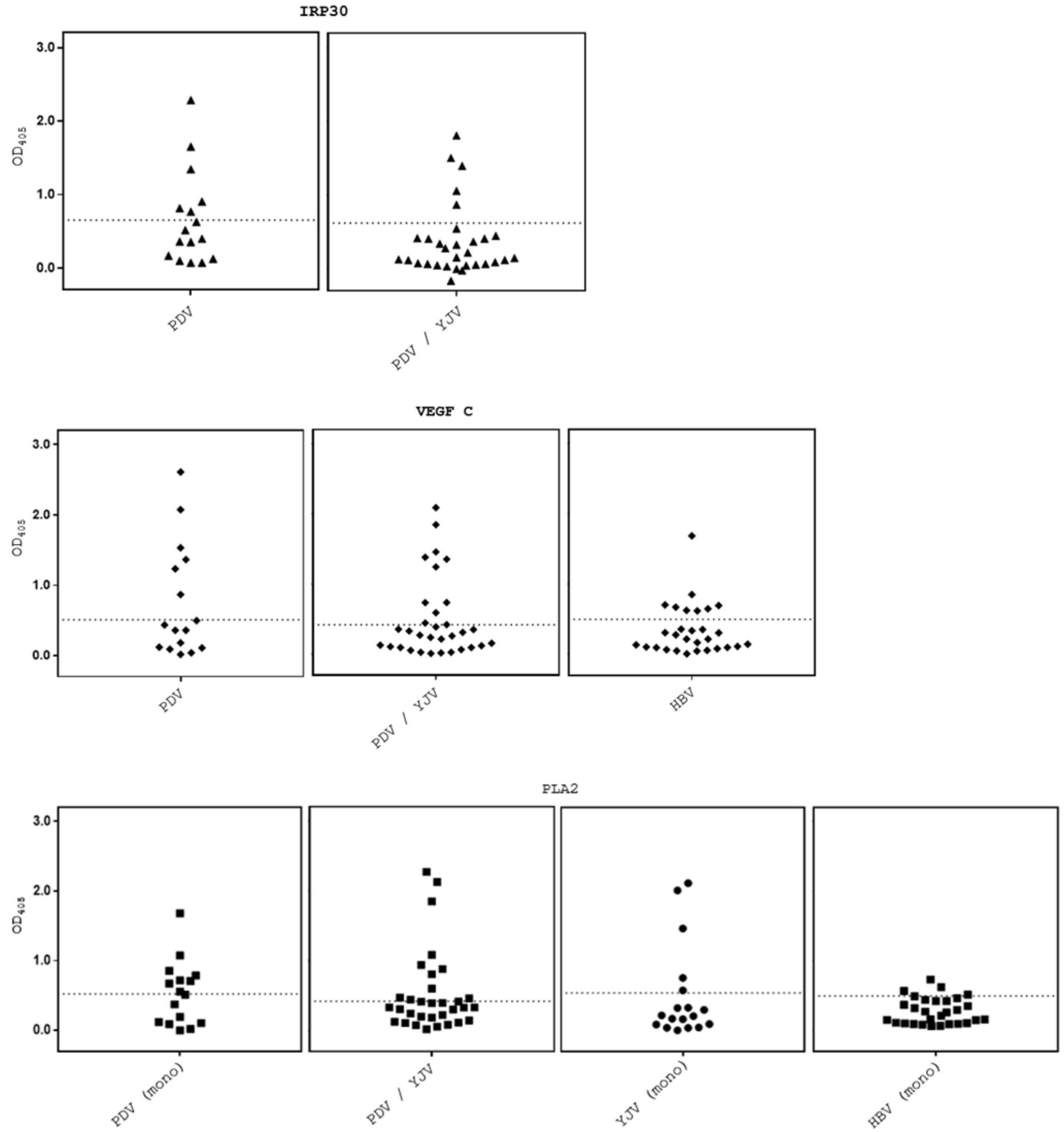

Figure S1. sIgE immunoreactivity of venom-sensitized patients to recombinant IRP30, VEGF C and PLA2 measured by ELISA. Positive and negative patients are separated by a cut-off (dotted line). Absorbance at 405 nm measured in

PDV ($n = 16$) mono-, PDV / YJV ($n = 31$) double-, YJV ($n = 18$) mono- or HBV ($n = 29$) mono-sensitized patients (left to right). PDV, *P. dominula* venom mono-sensitized patients; PDV/YJV, *P. dominula*/*V. vulgaris* venom double-sensitized patients; YJV, V. vulgaris venom mono-sensitized patients; HBV, *A. mellifera* mono-sensitized patients.

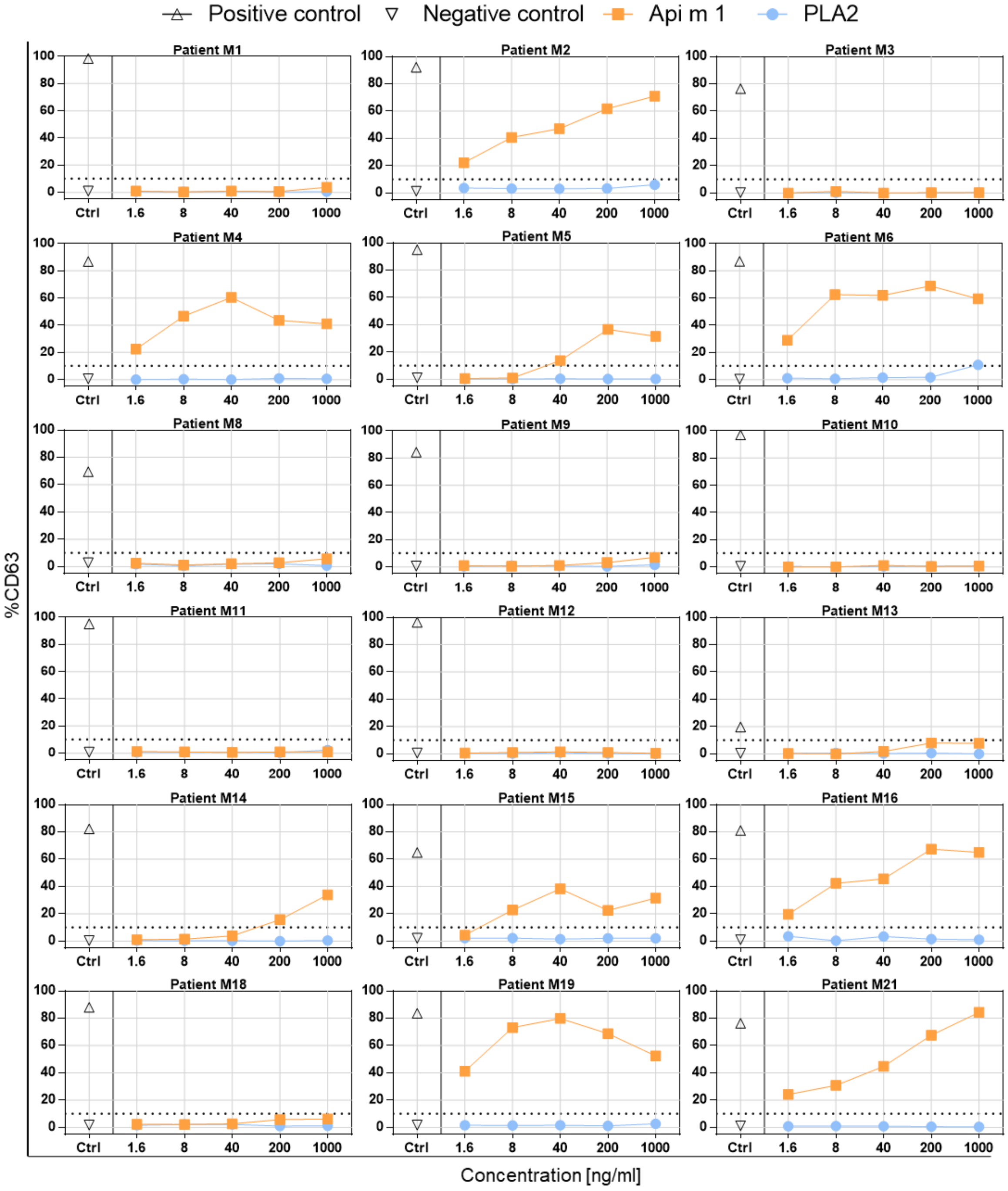

Figure S2. Increase of CD63 on basophilic cells of individual patients from greater Munich area upon stimulation with increasing doses of Api m 1 or PLA2.

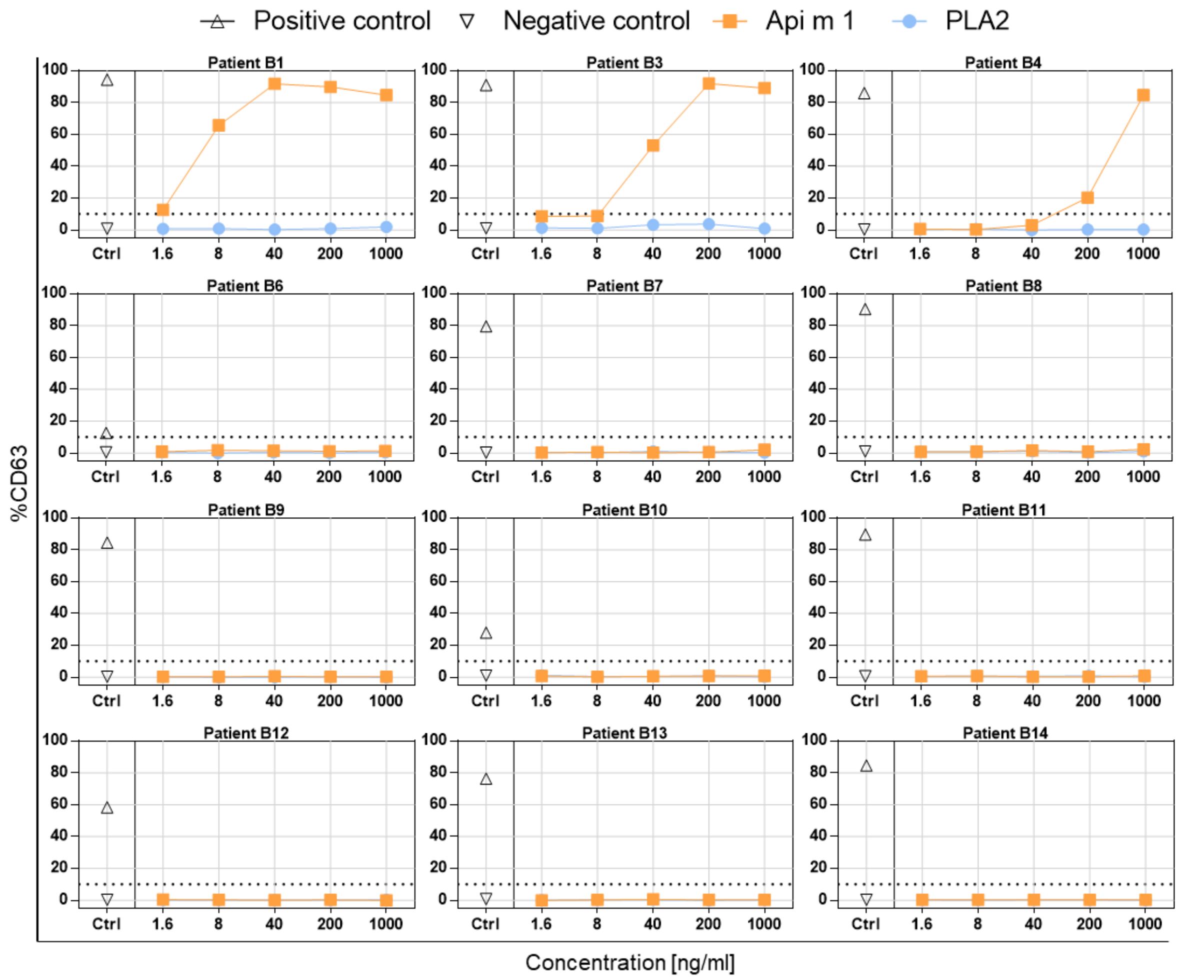

Figure S3. Increase of CD63 on basophilic cells of individual patients from greater Barcelona area upon stimulation with increasing doses of Api m 1 or PLA2.
